# The effect on cardiovascular risk factors of migration from rural to urban areas in Peru: PERU MIGRANT Study

**DOI:** 10.1186/1471-2261-9-23

**Published:** 2009-06-08

**Authors:** J Jaime Miranda, Robert H Gilman, Héctor H García, Liam Smeeth

**Affiliations:** 1 Department of Epidemiology and Population Health, London School of Hygiene and Tropical Medicine, London, UK; 2 Department of Medicine, School of Medicine, Universidad Peruana Cayetano Heredia, Lima, Peru; 3 Department of International Health, Johns Hopkins Bloomberg School of Public Health, Baltimore, MD, USA; 4 Unidad de Investigación en Enfermedades Parasitarias del Sistema Nervioso Department of Microbiology, School of Sciences, Universidad Peruana Cayetano Heredia, Lima, Peru; 5 Área de Investigación y Desarrollo, A. B. PRISMA, Lima, Peru; 6 Unidad de Cisticercosis, Departamento de Enfermedades Transmisibles y Neuropediatría, Instituto Nacional de Ciencias Neurológicas, Lima, Peru

## Abstract

**Background:**

Mass-migration observed in Peru from the 1970s occurred because of the need to escape from politically motivated violence and work related reasons. The majority of the migrant population, mostly Andean peasants from the mountainous areas, tends to settle in clusters in certain parts of the capital and their rural environment could not be more different than the urban one. Because the key driver for migration was not the usual economic and work-related reasons, the selection effects whereby migrants differ from non-migrants are likely to be less prominent in Peru. Thus the Peruvian context offers a unique opportunity to test the effects of migration.

**Methods/Design:**

The PERU MIGRANT (PEru's Rural to Urban MIGRANTs) study was designed to investigate the magnitude of differences between rural-to-urban migrant and non-migrant groups in specific CVD risk factors. For this, three groups were selected: Rural, people who have always have lived in a rural environment; Rural-urban, people who migrated from rural to urban areas; and, Urban, people who have always lived in a urban environment.

**Discussion:**

Overall response rate at enrolment was 73.2% and overall response rate at completion of the study was 61.6%. A rejection form was obtained in 282/323 people who refused to take part in the study (87.3%). Refusals did not differ by sex in rural and migrant groups, but 70% of refusals in the urban group were males. In terms of age, most refusals were observed in the oldest age-group (>60 years old) in all study groups. The final total sample size achieved was 98.9% of the target sample size (989/1000). Of these, 52.8% (522/989) were females. Final size of the rural, migrant and urban study groups were 201, 589 and 199 urban people, respectively. Migrant's average age at first migration and years lived in an urban environment were 14.4 years (IQR 10–17) and 32 years (IQR 25–39), respectively.

This paper describes the PERU MIGRANT study design together with a critical analysis of the potential for bias and confounding in migrant studies, and strategies for reducing these problems. A discussion of the potential advantages provided by the case of migration in Peru to the field of migration and health is also presented.

## Background

Much of the burden associated with NCDs are the result of environmental and lifestyle factors including tobacco consumption and decreased physical activity, and are preventable [1]. Despite this wealth of information available in the developed world, it is also clear that contexts are different – for example the impact of tobacco on mortality differs by geographical region [2] – and that there is an important research gap between developing and developed countries on these issues [3].

NCDs kill people at economically and socially productive ages and kill them mostly in the developing world: 80% of chronic disease deaths occur in LMIC [4]. As part of this growing concern with NCDs in LMIC [5], "grand challenges" for research and policy in this area highlights the need to study the impact of poverty and urbanization on NCDs [6]. Urban areas of developing countries are growing much faster [7], and their populations are larger [8], and thus, it becomes imperative to more fully understand the impact of migration on health in these settings.

Migration studies have provided informative findings related to the evaluation of morbidity and mortality patterns as well as risk factors associated to specific conditions amongst migrant and non-migrant groups [9]. Most of these findings, however, have been usually derived from the perspective of international migration. As such, most migrant studies' findings may bias the understanding of the impact of migration in LMIC where most migration follows a rural-to-urban pattern.

The picture is complex, since the effect of migration on a particular outcome varies according to who is migrating, when they migrate, where they migrate from, where they migrate to, and what health outcome is measured [9]. Migration is further complicated by the fact that it is not necessarily a random process; the "selection of migrants" and the "healthy migrant effect" -or, in some circumstances, the unhealthy migrant effect – may influence health and disease risk [10]. Such concern with migrant's selection bias dates back to 1938 [11]. Migration due to economic reasons poses additional difficulties in interpreting studies, since those with better health or socio-economic status could be the ones more likely to "afford" to migrate. It could, therefore, be argued that migrant groups are self-selected groups.

Relatively few studies have addressed the impact of rural-to-urban migration on CVD outcomes in LMIC, e.g. the Kenyan Luo migration study [12] and the Yi People Study in China [13], but these were conducted a few decades ago. Another limitation is that migrants were evaluated within a short-time frame, usually within 6-months after migration, and long-term assessments of the impact of migration are thus not available.

Peru offers a unique opportunity to assess the impact of migration on health. The patterns of migration in Peru changed dramatically during the political violence that occurred in the 1970–1990's period, where approximately 70,000 deaths occurred -79% of them in rural areas [14] – together with large amounts of displaced people – approximately 120,000 displaced families [15]. Thus, it could be argued, the mass-migration seen in Peru from 1980s onwards was largely driven by the need to escape from politically motivated violence rather than only a migration for economic reasons: the migrants were not simply a small self-selected atypical group.

Thus, the PERU MIGRANT study, together with other ongoing migrant research initiatives in India [16] and Cameroon [17], aims to provide up-to-date evaluations of the impact of migration on various health outcomes. The study design of the PERU MIGRANT study, response rates, analysis of responders versus non-responders and ascertainment of migration exposure are reported in this paper.

## Methods/Design

### Objectives

It was hypothesized that the risk of CVD increases following migration from rural to urban areas in Peru. The aim of this study was to ascertain whether or not differences exist in cardiovascular risk profiles between migrants and non-migrants as well as the magnitude of such differences. The following research questions were considered:

i) Is there a difference in specific CVD risk factors in the rural-to-urban migrant group compared to those who did not migrate?

Additionally, does the pattern of CVD risk factors in the migrant population vary by:

ii) length of residence in urban environment?

iii) lifetime exposure to urban environment?

iv) age at first migration?

### Study design

Cross-sectional survey of three population-based groups: i) Rural, people born in Ayacucho who have always have lived in a rural environment (n = 200); ii) Rural-to-urban migrants, people born in Ayacucho who migrated from rural to urban areas and currently living in Lima (n = 600); and, iii) Urban, who have always lived in an urban environment, that is people born and currently living in Lima (n = 200).

### Setting

Lima, Peru's capital, and Ayacucho. Ayacucho, an Andean department, was one of the most severely affected areas during this period of violence [18] – more than 50% of all deaths occurred in Ayacucho [14]. For the period 1988–1993, 50.7% of the total emigrants from Ayacucho moved to Lima, making Ayacucho the leading source of migrants to Lima [19].

**Figure 1:**
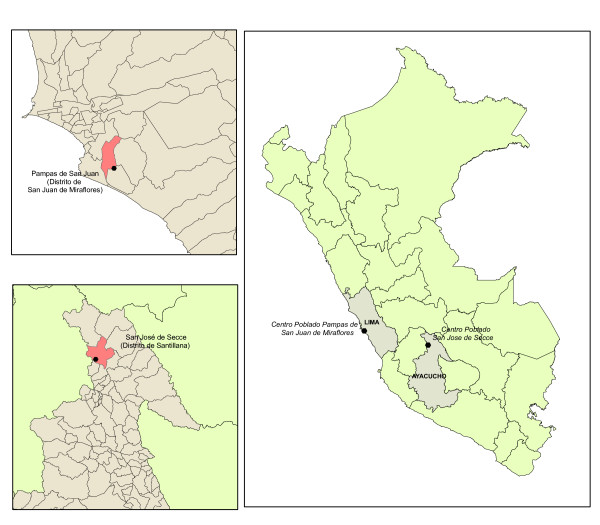
**Map of Peru and location of study sites in Lima and Ayacucho**.

The village of San Jose de Secce, located in the Santillana district, Huanta province in Ayacucho was selected as the rural study site (Figure 1). The area called "Las Pampas de San Juan de Miraflores" in Lima, was selected as the urban area for the study. Both urban and rural-urban migrant participants were selected from the Pampas de San Juan de Miraflores area, a periurban shantytown in the south of Lima.

### Participants

A single-stage random sampling method was used in all groups. In the case of San Jose de Secce in Ayacucho, a census was conducted in mid 2007 to identify all adult population permanently living in the area. The sampling frame for the urban group was derived from the local census, conducted in year 2000. All those who reported to have been born in Lima in the 2000 census and currently living permanently in the recorded address were considered eligible for the study. In the case of the rural-to-urban migrant group, the same 2000 census was updated in 2006 to identify all those who referred to have been born in the department of Ayacucho and were currently living in Lima.

For all study groups, individuals from both sexes aged 30 years-old and over, permanently living in their residence were considered eligible. Pregnant women and those with mental disorders that impair survey completion were excluded. Participant's selection was stratified by age-groups and sex to ensure sufficient number of people in each stratum.

### Study variables

#### Exposures

The primary exposure was migration from a rural to an urban environment, defined by study group, i.e. rural, rural-to-urban migrant and urban groups.

To address research questions ii, iii and iv, the migrant group was subsequently divided by length of residency in an urban area, lifetime exposure to an urban area, and age at first migration.

#### Outcomes

Primary outcomes: blood pressure, prevalence of hypertension, BMI, WHR, prevalence of obesity, fasting glucose, prevalence of diabetes, total cholesterol and lipoprotein profile.

Secondary outcomes: behavioral risk factors – alcohol consumption, smoking status-, inflammation markers – CRP, fibrinogen-, insulin resistance and metabolic syndrome.

### Data collection

Questionnaires were constructed after checking relevant work for the study, including the WHO STEPS [20]. For the fieldwork, the questionnaires were prepared and piloted in Spanish [see Additional File 1]. The Spanish versions of the DHS's Household Assets questionnaire [21] and CDC's Behavioral Risk Factor Surveillance System Survey Questionnaire [22] were also reviewed as reference instruments. In addition to the full survey, a rejection form was also elaborated for non-responders.

A team of community health workers with previous fieldwork experience on household visits was trained to enroll participants and to conduct the questionnaires. All assessments were made at the study coordinating centre by trained personnel.

#### Demographic and socioeconomic survey

Age, sex, contact details, place of birth, and proxies of current socioeconomic position – educational level, household income, number of people per room and asset's possession [23,24] – as well as paternal and maternal education levels attained were measured. Socioeconomic information was aggregated into deprivation indexes to provide an individual's current socioeconomic deprivation index [25-29].

#### Migration survey

Information about place of birth (rural or urban), age at first migration, age at arrival in Lima, education level at first migration and years lived in urban area were gathered. Lifetime exposure to urban area will be calculated as number of years lived in an urban area divided over current age, and expressed as percentage.

#### Risk factor survey

Frequency of alcohol consumption in the last year, volume of alcohol consumption, frequency of hangover in the last month, smoking status (never, former and current), from an adapted version of the WHO STEPS questionnaires [20], were recorded.

#### Clinical examination and measurements

Total and sitting height, measured to the nearest 0.1 cm using a stadiometer and standard stools, and weight, to be measured with the individual wearing light clothes to the nearest 0.05 Kg using SECA 940 electronic scale, were part of the examination.

Skinfolds were measured in triplicate in each measurement site (biceps, triceps, subscapular and suprailiac) to the nearest 0.2 mm using a Holtain Tanner/Whitehouse Skinfold Calliper . Waist circumference, measured in triplicate at the midpoint between the lower rib and the iliac crest, and hip circumference, measured in triplicate at the point yielding the maximum circumference over the buttocks were also included. Waist and hip measurements were made in the horizontal plane, while the participants were standing, using a tape measure to measure to the nearest 1 cm. For skinfolds, waist and hip circumference, the average of three measurements was calculated and used in the analysis.

SBP and DBP were measured using appropriate cuffs for arm circumference in the sitting position using the right arm, supported at chest level. Three measurements, at least 5 min apart using an oscillometric device (Omron M5-i, Omron, Japan) previously validated for adult population [30], were made. Mean of the last two SBP and DBP measurements were used for the analysis. Hypertension was considered as SBP ≥ 140 mm Hg or DBP ≥ 90 mm Hg, or self report of physician diagnosis and currently receiving antihypertensive medication [31,32].

#### Laboratory measurements

All laboratory assessments were performed by trained personnel on venous samples taken in the morning after a minimum of 8 hours fast.

Total cholesterol, triglycerides and HDL were measured in serum. In individuals with triglycerides below 400 mg/dL, LDL was calculated using the Friedewald equation [33,34], in mg/dL: LDL = total cholesterol - HDL - (0.2 × triglycerides). In individuals with triglycerides greater than 400 mg/dL, LDL was measured in serum. CRP and fibrinogen, inflammation markers, were also measured in serum and plasma, respectively.

Fasting glucose, fasting insulin and glycated hemoglobin were measured in plasma, serum and whole blood, respectively. Diabetes was considered as fasting glucose ≥ 126 mg/dL (or ≥ 7 mmol/L) [35] or self report of physician diagnosis and currently receiving antidiabetic medication. IR was calculated using HOMA calculator (Oxford Centre for Diabetes, Endocrinology & Metabolism, Diabetes Trials Unit, ) [36] and excluding those with diabetes.

#### Other instruments

The data collected will allow the classification of presence or absence of metabolic syndrome using various definitions [35,37-39]. In addition, the 12-item General Health Questionnaire [40] for mental health, a social capital instrument previously validated in Peru [41], and an acculturation scale [42] were also applied. Rose angina's and claudication questionnaire were also used to provide information about markers of sub-clinical CVD including peripheral artery disease.

### Study size

Power calculations were derived using conservative estimates of the prevalence of major risk factors in the areas of Huaraz (urban, Andes) and Ingeniería (urban, Lima) from preliminary work in Peru [43]. The study target was to recruit a total of 1000 people, i.e. 200 people in each of the rural and urban groups and 600 migrants.

Comparing the Lima with the Andes group, with 200 people in each group, the study had 80% power or greater at the 5% significance level to detect a difference in the prevalence of the following: hypertension 33% versus 19.5%, power 0.84; and, diabetes 7.6% versus 1.3%, power 0.81.

### Statistical methods

The rural group was used as the reference category for the main analyses. The migration variable will be subsequently sub-divided by length of residence in urban environment, lifetime exposure to urban area as a proportion of current age, and age at first migration, and the lowest categories in each of these will serve as baseline for comparisons.

For CVD-related outcomes, only data from all individuals who complete the study were used in the analysis. Participants who completed the study were defined as those with completed questionnaires, clinical measurements and laboratory analyses.

#### Descriptive analysis

For general description of data, frequency analyses will be calculated as number (percentages), mean (± SD) or median (IQR) when appropriate. Continuous non-normally distributed variables will be log transformed if necessary expecting that such logarithm transformation will lead to normal or near normal distributions. Age- and sex-adjusted arithmetic means (± SD) or geometric means (ratios) [44,45] will be presented. In the case of age, since the study-design only included participants from 30 years-old or more, a mid/centre age point will be used such that age 45 years-old will be considered as the baseline in all age-adjustments when applicable.

#### Multivariable analysis

Multivariable logistic regression and linear regression will be used for categorical and continuous outcomes respectively. Adjustment for potential confounding will be made in a step-wise approach. Adjustment for treatment effects in specific continuous outcomes, e.g. antihypertensive therapy on blood pressure outcomes, will be pursued using censored normal regression [46]. In categorical outcomes, odds ratios and 95% CI will be calculated using logistic regression. Prevalence ratios will also be calculated as indicated elsewhere [47].

#### Standardised mean differences

In the case of continuous outcomes, SMD will be chosen because of its advantage to interpreting results of continuous data measured with different scales or units, facilitating comparisons of difference sizes for individual measures. The Cochrane Collaboration has defined SMD as "the difference in means between two groups, divided by the pooled standard deviation of the measurements" and suggests that "the value of a SMD thus depends on both the size of the effect (the difference between means) and the standard deviation of the outcomes (the inherent variability among participants)" [48].

Due to the lack of units, SMD allows for comparison of magnitude of differences across various risk factors. In terms of the interpretation of SMD, the Cochrane Collaboration indicates that "rules of thumb exist for interpreting SMD (or 'effect sizes')... 0.2 represents a small effect, 0.5 a moderate effect, and 0.8 a large effect [48,49]".

### Ethics

Ethical approval for this protocol was obtained from ethics committees at Universidad Peruana Cayetano Heredia in Peru and London School of Hygiene and Tropical Medicine in the UK. The purpose of the study was explained to each of the study participants and informed consent was obtained, following international standards for ethical research in developing countries [50,51] [see Additional File 2 and 3].

## Results

### Completeness of data

Information was collected from a total of 994 individuals, and 24/944 (2.4%) had some degree of incomplete information in any of the eight study's modules (Table 1). Being the cardiovascular information the main outcomes of this study, only participants with completed questionnaires (those people who provided some information to all sections of the socioeconomic, migration and risk factors surveys), clinical measurements and laboratory analyses were considered as those who completed study, totaling 989 participants with completed cardiovascular data.

**Table 1:** Survey completeness according to study's modules

**Study's modules or questionnaires**	**Number of participants with incomplete modules**
	n = 944

1. Clinical measurements and laboratory analyses*	4 (0.4%)

2. Socioeconomic questionnaire*	1 (0.1%)

3. Migration questionnaire*	1 (0.1%)

4. Risk factor questionnaire*	3 (0.1%)

5. Physical activity questionnaire	4 (0.4%)

6. Acculturation questionnaire	3 (0.1%)

7. Social capital questionnaire	3 (0.3%)

8. Mental health questionnaire	16 (1.7%)

9. Rose Angina & claudication questionnaire	4 (0.4%)

Missing one module only	12 (1.2%)

Missing two modules	6 (0.6%)

Missing three modules	4 (0.4%)

Missing seven modules	1 (0.1%)

Missing eight modules	1 (0.1%)

Missing any module	24 (2.4%)

Notes: Only data from individuals who completed the modules marked with * were considered as participants with completed cardiovascular data (total 989/994). It is expected that missing data will exists in certain specific questions, measurements or laboratory tests for various reasons, but this did not disqualify the individual's information to be used on the data analysis process.

### Response rates

**Figure 2:**
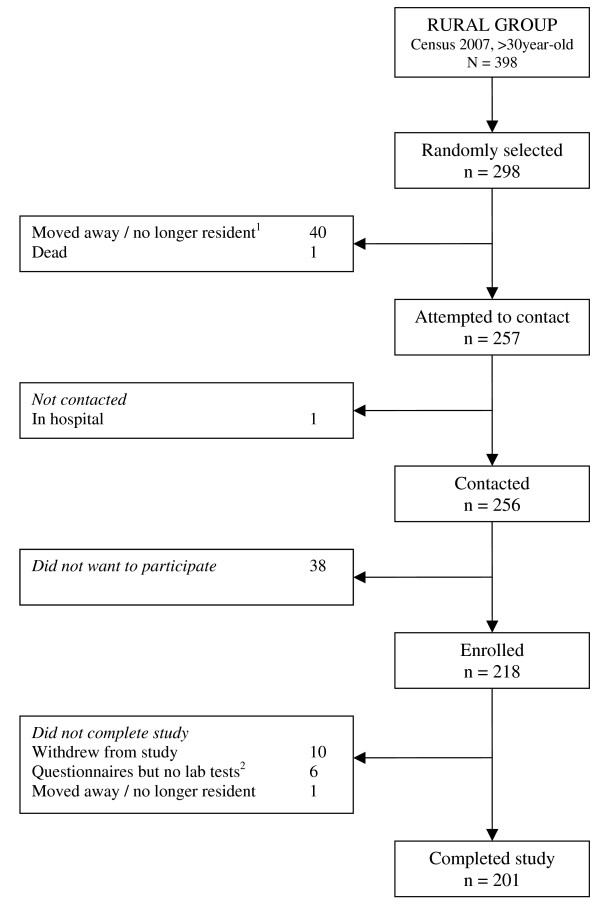
**Study participants' flowchart, rural group**. ^1^ Moved away / no longer resident was defined as those people who no longer live in the given address and moved to another area or are continuously living outside the area of study (e.g. house maids and security guards working and living full-time on employer’s houses/properties). This definition applies for all study groups. ^2 ^Questionnaires completed, but no laboratory tests were done because required sample size was reached and limited funds.

**Figure 3:**
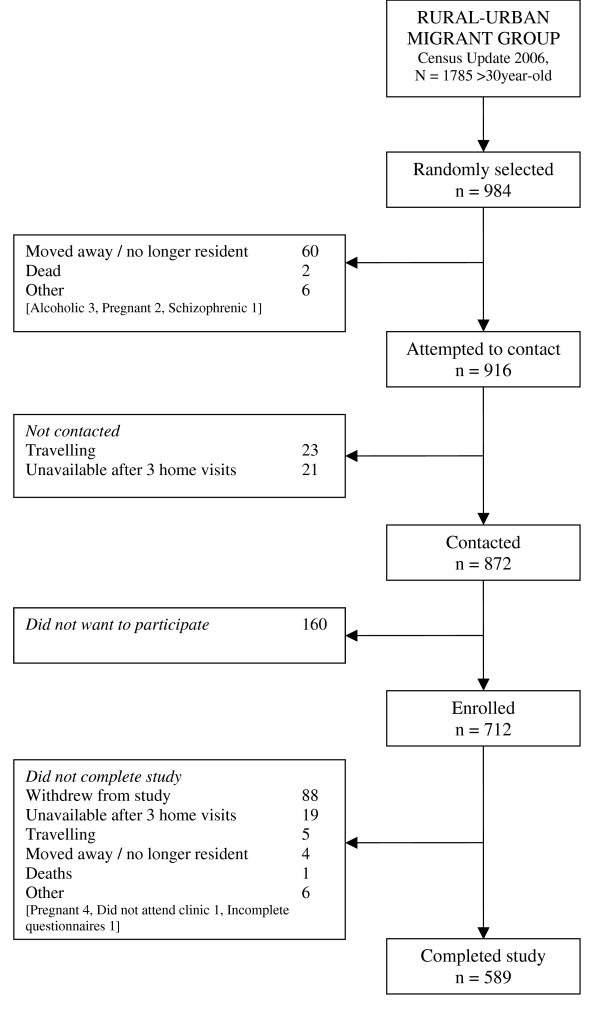
**Study participants' flowchart, rural-to-urban migrant group**.

**Figure 4:**
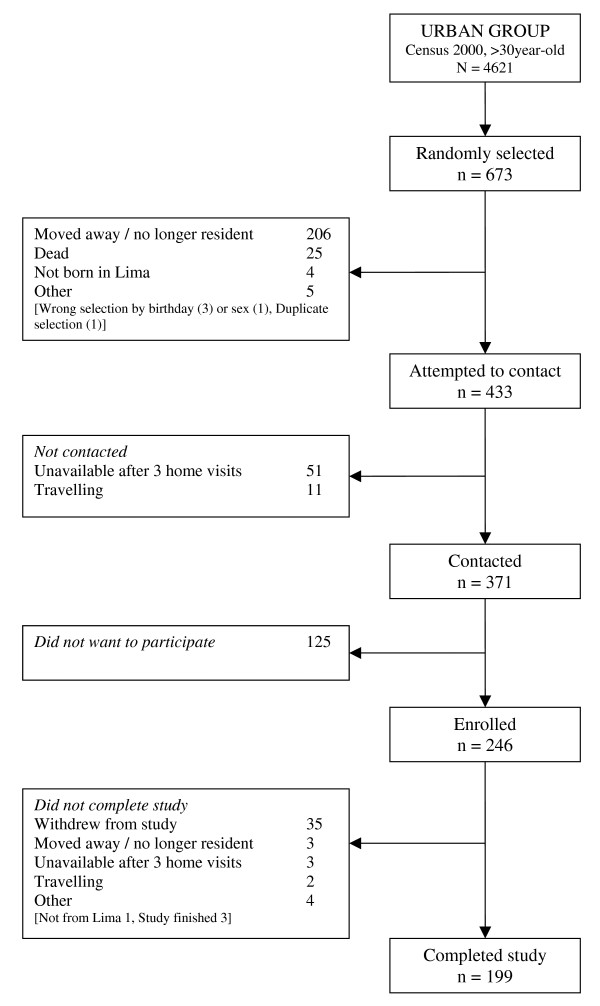
**Study participants' flowchart, urban group**.

The final total sample size achieved was 98.9% of the target sample size (989/1000). Of these, 52.8% (522/989) were females. Overall response rate at enrolment was 73.2% and overall response rate at completion of the study was 61.6%. Response rates were higher in the rural group and lower in the urban group (Table 2). Following STROBE recommendations for reporting of observational studies [52], Figure 2, 3 and 4 show numbers of individuals at each stage of study and sample attrition in each of the study groups.

**Table 2:** Response rates in study groups

	Response rate at enrolment*		Response rate at completion of study**		**Difference**
	n	%	n	%	%

Rural	218/257	84.8%	201/257	78.2%	6.6%

Migrant	712/916	77.7%	589/916	64.3%	13.4%

Urban	246/433	56.8%	199/433	46.0%	10.9%

Total	1176/1606	73.2%	989/1606	61.6%	11.6%

Notes:

* Response rate at enrolment = enrolled study/attempted to contact

** Response rate at completion of study = completed study/attempted to contact

### Non-responders

**Figure 5:**
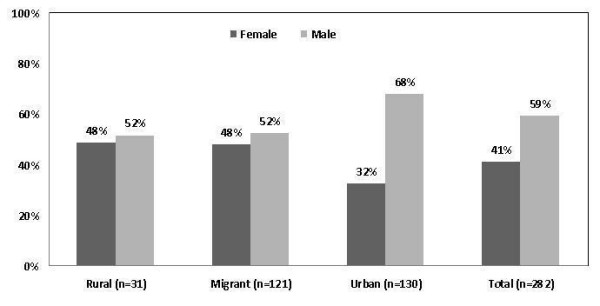
**Distribution of refusals by gender in each study group among the 282/323 non-responders who completed a rejection form**.

**Figure 6:**
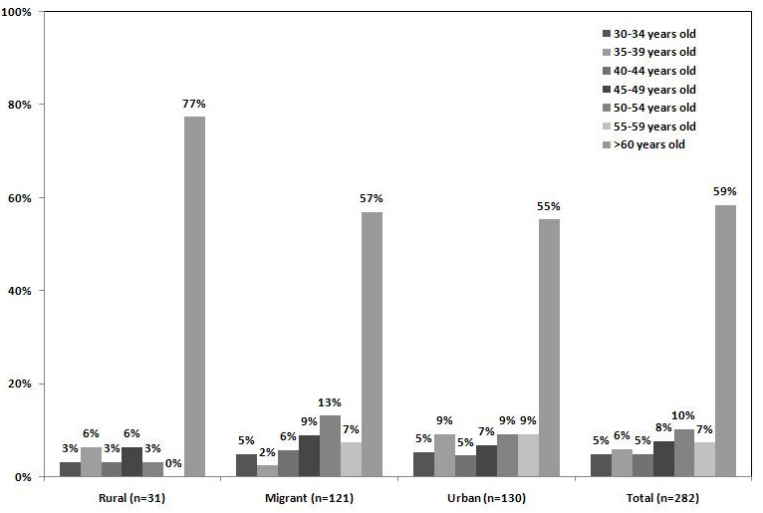
**Distribution of refusals by age in each study group among the 282/323 non-responders who completed a rejection form**.

A short rejection form survey was applied to those who refused to take part in the study gathering basic demographic and socioeconomic information as well as smoking status, previous diagnosis of hypertension and diabetes, current treatment for hypertension, age at migration and reasons for migration. This form was obtained in 282 people of the total 323 who refused to take part in the study (87.3%). Male non-responders were more likely to complete a rejection form (167/282, 59.2%). Among the different exposure groups, the proportions of non-responders providing a rejection form were: rural 31/282 (11%) migrants 121/282 (42.9%) and urban 130/282 (46.1%). A breakdown, by age and sex categories and study group are presented in Figure 5 and 6. A high proportion of refusals were observed in males in the urban group. In terms of age, most refusals were observed in the oldest age-group (>60 years old) in all study groups. The potential bias that these age and sex differences could have exerted in the main study are controlled, as the final population studied included similar proportions of sex and age strata.

Table 3 shows the different reasons provided for refusal to take part in the study. This was an open-ended question and responses were aggregated into four main categories. The most common reason for refusal was accessibility to social security insurance or access to medical check-ups in the public health system if necessary, thus not needing to take part in the study to benefit from the free evaluation provided. The next category for refusal was unwillingness to take part in the study, followed by logistical circumstances and finally health status.

**Table 3:** Reasons for refusing participation in the study

	**Rural**	**Migrant**	**Urban**	**Total**	Proportion*
	**n**	**n**	**n**	**n**	**%**

**Access to health care**					

Total	0	46	46	92	32.6%

Have social security insurance	0	26	33	59	20.9%

Have access to medical check-ups if wanted/needed	0	20	13	33	11.7%

**Unwillingness to participate**					

Total	0	43	24	67	23.8%

Distrust (e.g. disclosure of personal details, signature)	0	13	12	25	8.9%

"Do not want to participate" statement	0	11	7	18	6.4%

Did not want blood samples to be taken	0	12	4	16	5.7%

Negativism from relative of selected participant	0	7	1	8	2.8%

**Logistical**					

Total	1	17	32	50	17.7%

Time constraints due to working	0	6	12	18	6.4%

Time constraints, unspecified	0	5	13	18	6.4%

Travelling	0	4	1	5	1.8%

Other**	1	2	6	9	3.2%

**Health status**					

Total	0	16	14	30	10.6%

Recent laboratory test done, no results provided	0	9	5	14	5.0%

Sickness, non-CVD	0	2	5	7	2.5%

Recent laboratory test done, negative for CVD risk factors	0	1	3	4	1.4%

Sickness, unspecified	0	3	0	3	1.1%

Sickness, CVD***	0	1	1	2	0.7%

Notes: Reasons for refusal shown in this table are not mutually exclusive and were aggregated using four categories. The same individual could have answer more than one option.

* This proportion is calculated using the total of responses as numerator and the total of non-responders (n = 282) as denominator

** Other category included the following reasons: 4 religious beliefs or ideologies, 3 fieldwork mistakes with appointments, 1 pregnant, 1 do not live permanently in the area

*** Sickness CVD included 1 case with diagnosed diabetes and 1 case with diagnosed hypercholesterolaemia. In both cases treatment status was not specified.

### Comparison between responders and non-responders

The short rejection form, albeit not available for all non-responders, allowed the evaluation of comparable information between those who refused and those who completed the study [see Additional File 4]. No major differences were observed between rural non-responders when compared to their counterparts who completed the study, but numbers of non-responders were small in the rural group. However, amongst the urban group, non-responders differed from those who completed the study in education level. More urban non-responders had completed secondary level education (70.3% vs. 56.6% in urban responders). No differences in self reported diagnosis of diabetes or hypertension were seen between response groups. In relation to migration indicators, non-responders migrant's median age at first migration was similar compared to responders. Both, individual socioeconomic reasons – studies or working reasons- and terrorism were listed amongst the two main reasons for migration in both responders and non-responders.

To what extent non-response would have exerted an impact on CVD risk in this study is less clear given the paucity of evidence from Peru. Predicting the direction of bias in terms of CVD risk remains difficult. A sensible approach is to differentiate non-responders within study groups, and then, separately, how these within-group biases might affect the comparison of interest between groups. Based on the results presented, it could be that in all three groups, in within-group comparisons, a small tendency for responders to be slightly poorer than non-responders was observed. Therefore, when aggregated, the overall bias in between-group comparisons might be quite small.

### Ascertainment of migration exposure

The need to confirm the ascertainment of exposure lies in the fact that the migrant population studied has to be comparable to the rural population, as indicative of exposure to the same rural environment. Alternative ways to confirm this can be achieved by individual's self-ascertainment of their place of origin as rural or urban; by mother tongue; and, if the pattern of migration from Ayacucho into Lima is well established.

#### Confirmation of migrant status by place of origin

All participants in all study groups were asked to describe their place of birth, and separately the type of location, i.e. village, town or city. Nearly 80% in both rural and migrant group reported being born in a rural area. In addition to this, the most common description of the type of place for these groups was town and village, which coincide with the rural classification self-ascertained before. In the same vein, 81% of urban participants reported being born in an urban area, and nearly 90% described the place where they were born as a city or as the Capital (Lima).

#### Confirmation of migrant status by mother tongue

Ayacucho, the place of origin of both migrant and rural population in this study, is largely an indigenous area where most people speak Quechua, an ancient Peruvian language. In Lima, the language most used is Spanish. All study's participants were asked to name the first language they learnt to speak. Over 85% of participants in both rural and migrant groups reported Quechua as their mother tongue.

#### Confirmation of Ayacucho-Lima migration pattern

Migration from Ayacucho into Lima, particularly fostered by the years during terrorism, has been previously described. It is relevant to confirm such assumption since it could well be the case that most of migrants move to Lima but only after spending considerable time in another rural or urban area. This could affect the ratio of lifetime exposure to a rural environment versus an urban one. Amongst migrants, reported number of years living in an urban environment was 32 years (SD ± 10.5, IQR 25–39). Average age at first migration was 14.4 years (SD ± 8.5, IQR 10–17). Average age when arrived into Lima was 15.5 years (SD ± 8.8, IQR 11–18). There was good correlation between age at first migration and age when arrived into Lima (correlation coefficient = 0.92, p = < 0.0001) thus indicating that migrants, who were born in Ayacucho, tend to move largely directly into Lima.

## Discussion

The PERU MIGRANT Study achieved good overall response rates, a high-degree of data completion and included careful consideration of the potential for bias introduced by non-responders. In addition to this, migration, the exposure of interest, was assessed using a range of techniques. The study provides a solid framework for research into the impact of migration on health by comparing migrant versus non-migrant populations.

Migration is one example of social and cultural change [10]. Urbanisation, aided by migration, is a major feature of today's world [5,7]. In this context, this and other migration studies become relevant and crucial tools to address the impact of such complex processes on health. This background served as the basis to address to what extent migration may have had an impact on CVD risk factors in Peruvian population.

Findings from this study will provide a much clearer panorama, previously unknown, of the health profile of rural-to-urban migrants within Peru in relation to CVD risk factors. This information in turn, will prove useful for the understanding of CVD in Peru, and to certain extent in other LMIC undergoing similar process. It is hoped that, later on, the information generated in this study can inform key policy- and decision-makers in the design and implementation of preventative strategies.

Much epidemiology in the real world, it can be contended, revolves around the quantification of known risk factors, which are likely to interact in ways that are not seen in other settings. This study, by defining the patterns of differences in CVD risk that may exist in migrant and non-migrant populations, will open the venue for the need for further assessments to identify key determinants for such differences.

### Strengths of the study

The PERU MIGRANT Study takes advantage from one unique context of long-term established migration in a LMIC by studying adequate comparison groups for migrants, in both rural and urban areas. Rural people included those living in the same area where migrants originated while urban people were the ones living in the areas where migrants established. These well-defined study groups will enable the evaluation of one group of interest, i.e. migrants, in relation to rural and urban counterparts providing a complete panorama of CVD risk profile. The classification of migrants in subgroups according to length of migration, lifetime exposure to urban environment and age at first migration – usually not reported in migrant studies – will expand the evaluation of the impact of migration.

Razum [53] outlined what would be the requirements of an "ideal" migrant cohort which includes a unique definition of "migrant" that considers duration of stay. Additionally, and ideally, participants would have to be enrolled before they migrate, studies should include the population of origin of immigrants and studies should be based on individual data collected over time to understand the determinants of the relation between migration and health [53]. As suggested by Razum [53], the PERU MIGRANT study considers duration of stay in an urban area enabling the assessment of long-term exposures to an urban environment. This study have the added value of evaluating full CVD risk factors, including blood traits, in a context of increased urbanization that closely resembles today's LMIC. The standard definitions used will enable comparability of data with other resources from LMIC.

It is also worth noticing that the response rates observed in the present study working in poor urban and rural communities are not so different from those of larger studies conducted in developed countries. To name a few, the UK National Women's Health Study had a response rate of 49% for Stage 1 (a total of 26,050 questionnaires were returned in this stage) and 73% for the more targeted Stage 2 [54]. The British Regional Heart Study 1975–2004 response rate was 78% [55-57]. The British Women's Heart and Health Study had a 59.8% response rate (a total of 4286 women of the 7173 invited) [58,59]. In the US, the Atherosclerosis Risk in Communities Study' response rate ranged from 46% to 67% in the communities studied [60]. Therefore, the response rates observed in this study were within or above the range of response rates of internationally recognized well-conducted observational studies.

### Limitations of this study

Selection bias remains an important concern in migrant studies [53] and this study may not be free from this limitation. This is basically a concern with population denominators whereby migrants studied as a proportion of those who remain in their place of origin or as a proportion of total migrants are not generally known.

Due to the unique circumstances of the Peruvian context, where a forced migration process occurred, it would be expected that a wide diversity and majority of people from the rural part of Ayacucho had strong pressures to migrate, and not only the better-off – biologically and socioeconomically – sectors of this population. Rural and urban control groups were defined a priory to match the rural area of origin of most migrants as well as their urban destination. In this sense, although unavoidable, this study has benefited from a less extreme type of selection bias amongst the migrant populations in comparison to other studies of the same nature.

One major determinant of CVD risk, diet, has not been included in this study. Diet has an impact on a number of specific traits to be analyzed in this study, including obesity-related risk factors and lipid markers. These two factors are important ones for any study of CVD risk. However, this study was set out to find out whether or not differences exist in a number of risk factors and will not be capable, limited by its design, to explain but to postulate why these differences may occur.

## Conclusion

The PERU MIGRANT Study achieved good overall response rates, a high-degree of data completion and included careful consideration of the potential for bias introduced by non-responders. In addition to this, migration, the exposure of interest, was assessed using a range of techniques. The study provides a solid framework for research into the impact of migration on health by comparing migrant versus non-migrant populations in a low-income setting.

## Abbreviations

BMI: Body mass index; CDC: Center for Disease Control and Prevention; CI: Confidence intervals; CRP: C-reactive protein; CVD: Cardiovascular diseases; DBP: Diastolic blood pressure; DHS: Demographic Health Survey; HDL: High-density lipoprotein cholesterol; HOMA: Homeostatic model assessment; IQR: Interquartile range; IR: Insulin resistance; LDL: Low-density lipoprotein cholesterol; LMIC: Low- and middle-income countries; NCDs: Non-communicable diseases; PERU MIGRANT: PEru's Rural to Urban MIGRANTs; SBP: Systolic blood pressure; SD: Standard deviations; SMD: Standardised mean differences; STROBE: Strengthening the Reporting of Observational Studies in Epidemiology; USA: United States of America; UK: United Kingdom; WHO: World Health Organization; WHO STEPS: WHO STEPwise approach to Surveillance;WHR: Waist-to-hip ratio.

## Competing interests

The authors declare that they have no competing interests.

## Authors' contributions

JJM conceived the study, conducted the study and wrote the initial draft of this manuscript. RHG and HHG participated in the design of the study and actively supported the fieldwork phase of the study. LS contributed to the design of the study, the coordination of the study and provided critical input during data analysis and interpretation of results. All authors read and approved the final manuscript.

## Pre-publication history

The pre-publication history for this paper can be accessed here:



## Supplementary Material

Additional file 1 **PERU MIGRANT Study questionnaire**. Instruments used for data collection, as summarised in Table 1. Click here for file

Additional file 2 **Spanish information sheet and consent form**. Spanish informed consent form used in the PERU MIGRANT Study. Click here for file

Additional file 3 **English information sheet and consent form**. English translation of the informed consent form used in the PERU MIGRANT Study. Click here for file

Additional file 4 **Characteristics of responders versus non-responders by study group**. Comparison between those who refused (non-responders) and those who completed the study (responders) in terms of current socioeconomic status, CVD risk profile and migration history. Click here for file
