# Supplementary material for: The effect on cardiovascular risk factors of migration from rural to urban areas in Peru: PERU MIGRANT Study
Source: BMC Cardiovasc Disord. 2009 Jun 8;9:23. doi: 10.1186/1471-2261-9-23 (PMC2701408; doi:10.1186/1471-2261-9-23)
Supplement: Additional file 1 — PERU MIGRANT Study questionnaire. Instruments used for data collection, as summarised in Table 1. [file 1471-2261-9-23-S1.doc]

**CUESTIONARIO PARA SUJETOS QUE RECHAZAN EL ESTUDIO**

**Sujeto ID**

Centro

Comunidad

Casa

**1. Razón de no-entrevista**

No disponible Rehusar Otro

**2. ¿La persona está dispuesta a completar el cuestionario?** No Si

**3. ¿Cuántas personas viven en su casa?** En total

**4. ¿Cuál es su edad?** Años

**5. Grado de instrucción del paciente**

1. No fue a la escuela 2. Primaria incompleta 3. Primaria completa

4.Secundaria incompleta 5.Secundaria completa 6.Estudios técnicos o superiores incompletos 7.Estudios técnicos o superiores completos 8. Rehusa

**6. ¿Ud. fuma actualmente?**  1. Si 2.No

**7. ¿Ud. consume alcohol usualmente durante los fines de semana?** 1. Si 2. No

**8. Historia médica del paciente**

**Nota:** Si no está seguro de uno o más de los siguientes ítems por favor marcar la casilla de desconocido.

Desconocido No Si

Diabetes   

Cualquier enfermedad cardiovascular    

Cáncer   

**9. ¿Le ha dicho algún médico que tiene usted la presión arterial alta o hipertensión?**

**1. Si 2. No**

# Si su respuesta es NO pasar a la pregunta 11

**10. Siendo diagnosticada de Hipertensión alta, Ud. recibe alguna medicación**

**1. Si 2. No**

**11. ¿Cuál es el material predominante de los pisos de su vivienda?**

1. Piso natural: Tierra / Arena 2. Piso rústico: Entablado 3. Piso cemento no acabado 4. Piso acabado: Parquet /Vinílicos /Losetas / Cemento acabado

**12. ¿Cuál es el material predominante de las paredes exteriores de su vivienda?**

1. Ladrillo 2.Adobe 3. Madera 4.Triplay 5. Estera 6. Otro

**13. ¿Cuál es el material predominante del techo de su vivienda?**

1.Concreto 2.Madera 3.Tejas 4. Calamina 5. Caña con torta de barro

6.Otro

14. **¿Vive Ud. en el lugar en que nació?**

1. Si 2.No

Si la respuesta es Si, terminar la entrevista.

15. ¿**Qué edad tenía cuando dejó el lugar?** Años

**16. ¿Cuál fue el motivo principal de su salida de su lugar de origen?**

1.Estudios 2.Trabajo 3.Amenaza de terrorismo 4.Temor al terrorismo

5.Maltrato, violencia familia**r** y/o terrorismo 6**.**Enfermedad  **7.**Matrimonio

8.Alquiler de vivienda en otro lugar 9. Alojamiento con familiares

10.Comprar terreno/mudarse a casa propia 11.Otro......................................

**17. Nombre del entrevistador:**

**--------------------------------------------------------**

**Nombres y Apellidos**
