# Supplementary material for: The effect on cardiovascular risk factors of migration from rural to urban areas in Peru: PERU MIGRANT Study
Source: BMC Cardiovasc Disord. 2009 Jun 8;9:23. doi: 10.1186/1471-2261-9-23 (PMC2701408; doi:10.1186/1471-2261-9-23)
Supplement: Additional file 2 — Spanish information sheet and consent form. Spanish informed consent form used in the PERU MIGRANT Study. [file 1471-2261-9-23-S2.pdf]

DOCUMENTO DE INFORMACIÓN Y FICHA DE CONSENTIMIENTO PARA UN ESTUDIO

Estudio: Efecto de la migración rural-urbana en los factores de riesgo para enfermedad cardiovascular en Perú
Instituciones: London School of Hygiene and Tropical Medicine (Londres, Reino Unido)
Universidad Peruana Cayetano Heredia (Lima, Perú)
Investigadores: Dr. Jaime Miranda, Dr. Liam Smeeth, Dr. Carlos Cáceres, Dr. Juan Lema

<u>Propósito del Estudio.</u>
Nos gustaría invitarlo a participar en este estudio cuyo propósito es determinar en la población condiciones que están relacionadas con enfermedades del aparato circulatorio, tales como obesidad, presión arterial elevada, presencia de diabetes y colesterol elevado.

Estamos invitando a personas que hayan nacido en el departamento de Ayacucho y que se mudaron a Lima durante los años de violencia política. Así mismo, estamos invitando a personas que no han migrado –personas nacidas y que viven actualmente en Lima y Ayacucho, respectivamente–, a fin de poder comparar las condiciones mencionadas arriba entre estos grupos de poblaciones. Esperamos hacer este estudio en un total de 1000 personas, entre Ayacucho y Lima. Antes de decidir si desea participar en este estudio, por favor lea este documento y discútalo con el entrevistador.

Actualmente se sabe que existe un riesgo incrementado de sufrir ataques al corazón (infartos de miocardio) o de "derrames cerebrales" (conocido médicamente como accidentes cerebro vasculares) en aquellas personas que tienen uno o mas de los siguiente diagnósticos: diabetes, de presión alta, de tener ciertos lípidos elevados (como el LDL colesterol y los triglicéridos) y obesidad.

En este estudio nos gustaría hacerle una entrevista breve para obtener información general relacionada a estas enfermedades, tomar medidas de talla, peso y cintura, y tomar una muestra de sangre para poder determinar mediante un estudio de laboratorio las condiciones que queremos estudiar. Nosotros guardaremos pequeñas cantidades de cada muestra de sangre en un congelador, para verificar en el futuro cualquier error en caso encontremos resultados inesperados durante el estudio. Así mismo, guardaremos una pequeña parte de sus muestras de sangre, para en el futuro, poder continuar los estudios comparando lo encontrado con otras poblaciones.

Este estudio es financiado por una organización caritativa llamada The Wellcome Trust.

<u>Procedimientos.</u>
Si usted decide participar en este estudio, nosotros:

- Le pediremos que firme la ficha de consentimiento adjunta.
- Le haremos preguntas acerca de usted y su salud, y mediremos su talla, peso y cintura.
- Le mediremos la presión arterial hasta en tres oportunidades
- Le pediremos que nos permita sacar una muestra de sangre, solamente una vez, equivalente a tres cucharaditas de te
- Haremos análisis en sangre, sin ningún costo para usted.
- Congelaremos y guardaremos una pequeña porción de cada muestra (con una etiqueta de identificación que no contendrá su nombre).
- Le devolveremos los resultados de sus estudios de sangre, para que usted cuente con una copia personal.

<u>Razones para no ser incluidos en el estudio.</u>
Usted no podrá unirse al estudio si es menor de 25 años.

<u>Beneficios.</u>
El principal beneficio de este estudio es que mucha gente no sabe si sufre de presión alta, diabetes o lípidos elevados. De suceder esto en su caso, usted podrá conocer si tiene algunas de estas condiciones y buscar ayuda especializada respectiva. Usted no recibirá ningún dinero por participar en este estudio.

<u>Riesgos e Incomodidades.</u>

Nada en este estudio representa un gran riesgo a su salud. La mayoría de preguntas que le haremos en esta visita son preguntas que le harían normalmente en un centro de salud. Responder a todas nuestras preguntas le tomará solamente unos cuantos minutos de su tiempo. Las pruebas adicionales serán realizadas usando las muestras de sangre. La obtención de la muestra de sangre tiene riesgos mínimos y se hace utilizando la misma técnica que usan los profesionales en los centros de salud y hospitales. Simplemente necesitamos su permiso para hacer estas pruebas en su sangre, y para hacerle preguntas acerca de usted y su salud.

Privacidad.
Para el propósito de este estudio, toda la información acerca de usted y los resultados de las pruebas que realicemos será recogida y guardada en una base de datos en computadoras protegidas con contraseñas. Incluso así, a usted no se le podrá identificar ya que se usarán números en vez de nombres en la base de datos. Sin embargo, con el propósito de poder reportar los resultados a los participantes, sus muestras podrán ser identificadas por el personal de laboratorio como es rutina con la pruebas de laboratorio. Los resultados de sus pruebas sólo serán de conocimiento del personal se laboratorio, del investigador principal, y de usted.

Participación.
Usted no tiene que participar en este estudio si no lo desea. Si usted decide no participar en el estudio, el cuidado que a usted y su familia se les brinda en el centro de salud o por cualquier otro doctor no será afectado de ninguna manera. Si decide no participar, no se harán las preguntas sobre su salud, ni las mediciones de peso y talla, ni se tomará la muestra de sangre.

Preguntas.
Puede preguntar acerca de cualquier cosa que no entienda, ahora o en el futuro. Usted puede preguntarle al personal del estudio, o puede llamar a los números telefónicos que aparecen al comienzo de esta ficha. Puede también hacer preguntas acerca de la ética del estudio al Comité Institucional de Ética de la Universidad Peruana Cayetano Heredia (teléfono 319-0005, anexo 2271).

Usted puede comunicarse con el investigador responsable de este estudio y las personas que participan en el momento que desee por cualquier motivo relacionado al mismo. Para ello, por favor sírvase llamar a los teléfonos indicados:

Contactos: Dr. Jaime Miranda, Investigador Principal 292 0999

Si firma su nombre abajo, significa que usted ha entendido el contenido de este documento y ha decidido participar de este estudio.

Firma del Participante: ……………………………………………………………………………….
Nombre del Participante: ……………………………………………………………………………….

Si el Participante no sabe leer, entonces necesitamos también la firma de un Testigo:
Firma del Testigo: ……………………………………………………………………………….
Nombre del Testigo: ……………………………………………………………………………….

Firma del Encuestador: ……………………………………………………………………………….
Nombre del Encuestador: ……………………………………………………………………………..

Firma del Investigador: ……………………………………………………………………………….
Toda esta ficha debe ser completada en la misma fecha, la cual debe estar especificada aquí:

Fecha: ……………………..Hora:……………………..

Nosotros le entregaremos una copia de este documento para sus registros.
