# Supplementary material for: The effect on cardiovascular risk factors of migration from rural to urban areas in Peru: PERU MIGRANT Study
Source: BMC Cardiovasc Disord. 2009 Jun 8;9:23. doi: 10.1186/1471-2261-9-23 (PMC2701408; doi:10.1186/1471-2261-9-23)
Supplement: Additional file 3 — English information sheet and consent form. English translation of the informed consent form used in the PERU MIGRANT Study. [file 1471-2261-9-23-S3.pdf]

INFORMATION SHEET AND CONSENT FORM FOR A STUDY

**Study:**	The effect on cardiovascular risk factors of migration from rural to urban areas in Peru
**Institutions:**	London School of Hygiene and Tropical Medicine (London, United Kingdom)<br>Universidad Peruana Cayetano Heredia (Lima, Peru)
**Investigators:**	Dr. Jaime Miranda, Dr. Liam Smeeth, Dr. Carlos Cáceres, Dr. Juan Lema

Purpose of the Study.

We would like to invite you to participate in this study aimed to determine those conditions related to diseases of the circulatory system such as obesity, high blood pressure, diabetes and high cholesterol levels among the population.

We are inviting people who have been born in the department of Ayacucho and moved to Lima during the years of political violence. Also, we are inviting people who have not migrated – people born and currently living in Lima and Ayacucho, respectively – in order to compare the conditions mentioned above between these population groups. We hope to carry out this study in a total of 1000 people, both in Ayacucho and Lima. Before making a decision about whether you wish to participate in this study, please read this document and discuss it with the interviewer.

It is now known that there is an increased risk of heart attack (myocardial infarction) or "strokes" (medically known as cerebrovascular accidents) in people who have been diagnosed with one or more of the following diseases: diabetes, high blood pressure, high levels of serum lipids (such as LDL cholesterol and triglycerides) and obesity.

In this study, we would like to make a brief interview to gather general information related to these diseases, take measurements of height, weight and waist, and take a blood sample to determine, through a laboratory study, the conditions we want to study. We will store small quantities of each blood sample in a freezer, to verify any error in the future, such as in the event that we find unexpected results during the study. Also, we will store a small part of your blood samples in order to be able, in the future, to continue with the studies, by comparing our findings with other populations.

This study is financed by a charity called The Wellcome Trust.

Procedures.

If you decide to participate in this study, we will:

- Ask you to sign the consent form enclosed.
- Ask questions about you and your health, and measure your height, weight and waist.
- Measure your blood pressure up to three times
- Ask you to allow us to take, only once, a blood sample equivalent to three teaspoons.
- Carry out a blood analysis, at no cost to you.
- Freeze and store a small portion of each sample (with an identification label that will not include your name)
- Give you the results of your blood tests, for your records.

Reasons for not being included in the study.

You may not participate in the study if you are under 25 years old.

Benefits.

The main benefit of this study is that many people do not know if they suffer from high blood pressure, diabetes or elevated serum lipid levels. Should this be your case, you will know if you have any of these conditions and be able to seek appropriate care. You will not be given any money for participating in this study.

Risks and Discomforts

Nothing in this study represents a great risk to your health. Most questions we will ask in this visit are questions that would be normally asked in a health centre. It will only take a few minutes of your time to answer all the questions. Additional tests shall be made using the blood samples. Blood samples are obtained with minimum

risk, and using the same technique used by professionals in health centres and hospitals. We just need your permission to carry out these tests on your blood and to ask questions about yourself and your health.

Privacy.

For the purposes of this study, all the information about yourself and the results of the tests carried out will be collected and stored in a database in password-protected computers. Even so, you will not be identifiable, as numbers will be used instead of names in the database. However, in order to be able to report the results to participants, your samples will be identifiable by the laboratory staff, as is customary with laboratory tests. Your test results will only be known by the laboratory staff, the main researcher and yourself.

Participation.

You do not have to participate in this study if you do not want to. If you decide not to participate in the study, care provided to you and your family in the health centre or by any other physician will not be affected in any way. If you decide not to participate, no questions about your health will be asked; no weight and height measurements will be made; and no blood samples will be taken.

Questions.

You may ask any questions about anything that is not clear, either now or in the future. You may ask the staff involved in the study or call the phone numbers shown at the beginning of this form. You may also ask questions about the study ethics to the Institutional Ethics Committee of the Universidad Peruana Cayetano Heredia (telephone 319-0005, ext. 2271).

You may contact the investigators responsible for this study and people taking part of it at any time, and for any reason related to this study. For such purpose, please call the following numbers:

Contact: Dr. Jaime Miranda, Principal Investigator 292 0999

By signing below, you agree that you have understood the contents of this document and decided to participate in this study.

Participant's signature: ……………………………………………………………………………….
Participant's name: ………………………………………………………………………………….

If the Participant is illiterate, then the signature of a Witness is also required:
Witness' signature: ……………………………………………………………………………….
Witness' name: ………………………………………………………………………………….

Interviewer's signature: ……………………………………………………………………………….
Interviewer's name: ………………………………………………………………………………….

Researcher's signature: ……………………………………………………………………………….
The whole form must be completed on the same date, which must be specified below:

Date: ……………………..Time:……………………..

You will be provided with a copy of this document for your records.
