# Supplementary material for: The effect on cardiovascular risk factors of migration from rural to urban areas in Peru: PERU MIGRANT Study
Source: BMC Cardiovasc Disord. 2009 Jun 8;9:23. doi: 10.1186/1471-2261-9-23 (PMC2701408; doi:10.1186/1471-2261-9-23)
Supplement: Additional file 4 — Characteristics of responders versus non-responders by study group. Comparison between those who refused (non-responders) and those who completed the study (responders) in terms of current socioeconomic status, CVD risk profile and migration history. [file 1471-2261-9-23-S4.doc]

Characteristics of responders versus non-responders

|  | **Non-responders** | | | **Completed study (responders)** | | |
| --- | --- | --- | --- | --- | --- | --- |
|  | **Rural** | **Migrant** | **Urban** | **Rural** | **Migrant** | **Urban** |
|  |  |  |  |  |  |  |
| **Socioeconomic** |  |  |  |  |  |  |
| Number of people living in the same household | n = 6 | n = 66 | n = 72 | n = 200 | n = 589 | n = 199 |
| Median (IQR) | 3.5 (3 - 4) | 6 (4 - 7) | 5 (3.5 – 6.5) | 5 (4 – 7) | 5 (4 – 7) | 5 (4 – 7) |
|  |  |  |  |  |  |  |
| Education level attained (n, %) | n = 6 | n = 67 | n = 74 | n = 201 | n = 588 | n = 198 |
| None | 0 | 9 (13.4%) | 2 (2.7%) | 68 (33.8%) | 59 (10%) | 2 (1%) |
| Primary incomplete | 2 (33.3%) | 18 (26.9%) | 3 (4.1%) | 64 (31.8%) | 124 (21.1%) | 11 (5.6%) |
| Primary complete | 3 (50%) | 7 (10.5%) | 4 (5.4%) | 30 (14.9%) | 99 (16.8%) | 23 (11.6%) |
| Secondary incomplete | 1 (16.7%) | 12 (17.9%) | 13 (17.6%) | 16 (8%) | 126 (21.4%) | 50 (25.3%) |
| Secondary complete or more | 0 | 21 (31.3%) | 52 (70.3%) | 23 (11.4%) | 180 (30.6%) | 112 (56.6%) |
|  |  |  |  |  |  |  |
| **Cardiovascular Risk Factors** |  |  |  |  |  |  |
| Current smoker* (n, %) | 1/6 (16.7%) | 8/66 (12.1%) | 12/75 (16%) | 11/201 (5.5%) | 59/589 (10%) | 40/199 (20.1%) |
| Diabetes diagnosis, self-report  (n, %)** | 0/6 | 0/67 | 5/75 (6.7%) | 0/201 | 14/589 (2.4%) | 9/199 (4.5%) |
| Hypertension diagnosis, self-report (n, %)** | 0/7 | 7/67 (10.5%) | 9/74 (12.2%) | 12/201 (6%) | 59/589 (10%) | 28/199 (14.1%) |
|  |  |  |  |  |  |  |
| **Migration history** |  |  |  |  |  |  |
| Age when left place of birth |  | n = 62 |  |  | n = 572 |  |
| Years, Average (±SD) |  | 18.7 (±13.7) |  |  | 14.7 (±9) |  |
| Years, Median (IQR) |  | 15 (10 – 22) |  |  | 14 (10 – 17) |  |
|  |  |  |  |  |  |  |
| Main reason for migration (n. %) |  | n = 67 |  |  | n = 590*** |  |
| Socioeconomic, individual |  | 53 (79.1%) |  |  | 384 (65.1%) |  |
| Terrorism |  | 9 (13.4%) |  |  | 114 (19.3%) |  |
| Socioeconomic, family |  | 0 |  |  | 5 (0.8%) |  |
| Family/partner |  | 3 (4.5%) |  |  | 84 (14.2%) |  |
| Other factors |  | 2 (3.0%) |  |  | 3 (0.3%) |  |
|  |  |  |  |  |  |  |

Notes:

* Current smoking status in the non-responders was evaluated as a Yes/No question. In the case of the ones who completed the study, current smoker was defined as someone who smoked more than 100 cigarettes in lifetime and last cigarette was less than 6 months ago.

** Diabetes and hypertension correspond to self-report only to enable a similar comparison across groups. These figures will differ from prevalences to be reported later because they do not include diagnosis based on blood pressure or glycaemia measurements.

*** Main reasons for migration were drawn from information gathered initially in the census.
